# Assessing prevalence, factors and health consequences and academic performance of undergraduate students with breakfast skipping during COVID-19 using statistical modeling: a cross-sectional study

**DOI:** 10.1007/s40519-024-01676-2

**Published:** 2024-08-16

**Authors:** Dler H. Kadir, Mahmood Fadhil Saleem, Yaseen Galali, Azhin M. Khudr, Holem Hashm Balaky, Hamed Hassanzadeh, Babak Ghanbarzadeh

**Affiliations:** 1 Department of Statistics, College of Administration and Economics, Salahaddin University-Erbil, Erbil, Iraq; 2 Department of Food Technology, College of Agricultural Engineering Sciences, Salahaddin University-Erbil, Erbil, Iraq; 3 Department of Nutrition and Dietetics, Cihan University-Erbil, Erbil, Iraq; 4 Department of General Science, Faculty of Education, Soran University, Soran, Kurdistan Region Iraq; 5 Mergasor Technical Institute, Medicine Plant, Erbil Polytechnic University, Erbil, Iraq; 6 Department of Food Science and Hygiene, Faculty of Para-Veterinary, Ilam University, Ilam, Iran; 7 Department of Food Science and Technology, Faculty of Agriculture, University of Tabriz, Tabriz, Iran; 8 Department of Food Engineering, Faculty of Engineering, Near East University, Mersin, Turkey

**Keywords:** Covid-19, Breakfast skipping, Statistical modeling, Undergraduates, Health consequences

## Abstract

The study was conducted in order to study breakfast skipping (BKS) frequency, factors associated with, health consequence and undergraduate students academic performance during Covid-19 pandemic as earliest studies focusing on this area. A cross-sectional study was carried out among 2225 of undergraduate students. The study was carried between the period of 15/1/2020 to 3/4/2020 using an online self-report Breakfast Eating Habit Survey (BEHS). The BEHS survey was divided into two sections. The first sections included sociodemographic information (gender, BMI, age, smoking, residency, parental education, family income, studying system and stage (public or private), and studying institution (university or institute) academic performance. The second part included questions regarding breakfast eating habits including frequency of skipping meals, factors related to BKS health consequences and types of snacks. Logistic regression is a common technique used for modeling outcomes that fall into the range of 1 and 0. For this purpose, a logistic regression was performed to find adjusted odds ratio and crude odds ratio. The results showed that the majority of participants were female (1238, 55.7%). Out of 2,224 students, 2059 are aged between 18 to 24 years. Most of the participants were from first level (26.5%), second level (32.8%), third level (17.6%) or the fourth level (21.3%). Over 92% of participants were single and about 68% came from families of medium income families. The statistical analysis showed that the odds of BKS is reduced among students who live in accommodation by 54% (odds ratio = 54%, CI (41–71%), *p* value = 0.000). It seems that students with low income and normal or higher BMI are more likely to skip breakfast more regularly. The odds of skipping breakfast among students with BMI of 18–24.9 is reduced by 41% (odds ratio = 59%, CI (27%-93%), *p* value = 0.027) and the odds of BKS is reduced among students with BMI of 25–29.9 by 45% (odds ratio = 55%, CI (31–95%). Additionally, students with medium or high incomes are more likely to skip breakfast as much as twofold in comparison with students with low income (medium income (odds ratio = 1.85, CI (1.08–3.17), *p*-value = 0.024), high income (odds ratio = 1.98, CI (1.12–3.51), *p*-value = 0.019). The most common reasons for skipping breakfast included include time constraint, not hungry, breakfast is not ready, afraid to be overweight and lack of appetite. The consequences of skipping breakfast were feeling hungry throughout the day, feeling tired, and not paying attention in class and low academic performance. To concluded, BKS during Covid-19 is more common among students with higher BMI, higher income and living in accommodation. The main reason is time constraint and the most common health problems are being tired and luck of attention.

## Introduction

Breakfast is one of the most important meals throughout the day. It can provide and fuel the body with required energy and micronutrients following a long night fasting [23]. Breakfast could also play a crucial role in improving body well-being and preventing some non-communicable chronic diseases [23]. In contrast, breakfast skipping (BKS) could cause many cognitive and health problems including lack of focus and low academic performance [19]. Furthermore, BKS might be difficult to compromise and could lead not just to ingest more calories and overeating [21], but also consuming less important micronutrients like vitamins, fibers, minerals and phytochemicals [25].

The year of 2019 was recognized by the emergence of Covid-19 and caused the impose of several changes in life style routines as a results of mandate lockdown and confinement [5, 20]. For instances, educational institutes were closed, classes become at-distance and/or online. These changes affected students eating behavior including more vegetable and fruits as well as snacks and meals. [6]. Studies have shown that snacks were consumed more by 52% [22] and breakfast by 5% [20]

Dietary habit and lifestyle are profoundly influenced by the spread of the Covid-19 particularly among youngsters. According to literature available, BKS is more prevalent during Covid-19 among youngsters than older adults [7], particularly among undergraduates which related several well-being compromising behavior [3, 18]. Consequently, it is more likely to lead to several negative health complications in short term.

Several aforementioned studies have connected BKS with increasing weight change, negative health consequences and academic performance. However, there is a shortage and more information is required to obtain an insight of the prevalence of BKS among large population of undergraduate students particularly in relation to health consequences, factors for skipping and academic performance during pandemics. Therefore, the aim of present study is to study BKS frequency, factors associated with, health consequence and undergraduate students academic performance during Covid-19 pandemic as earliest studies focusing on this area.

## Material and methods

### Participants and sample size

A descriptive cross-sectional study was carried out and sent to the undergraduate students in Kurdistan universities in a randomized sampling method. A pilot study was conducted to assess the validity and reliability of the questionnaire. Then, the questionnaire was reconstructed and rearranged accordingly.

The study was carried out among Kurdish students and a total of 2225 of undergraduate Kurdish students aged between 18–25 years old responded correctly. The study was carried between the period of 15/1/ 2020 to 3/4/2020 after emerging Covid-19. Due to the Covid-19 pandemic limited movement and to maximize student’s involvement, the snowball online form was distributed among the students.

### Breakfast Eating Habit Survey (BEHS)

A self-declared BEHS was designed based on the previous researchers with some modifications [24]. It was then translated it to Kurdish language to ease understanding. The evaluation BKS and/or regularity and factors related to over a period of seven days. The questionnaire was divided into two sections. The first sections included sociodemographic information (gender, age, smoking, residency, parental education, average monthly family income, studying system (public or private), studying institution (university or institute). The second part included questions regarding breakfast eating habits including number of skipping meals, snack foods, factors and health consequences related to BKS.

### Ethical approval and consent form

Ethical approval for University of Salahaddin-Erbil and consent from the students was attained before commencing this study. Students were ascertained that their personal information was voluntary and the information kept anonymous.

### Statistical analysis

In the current study, a Chi-square test of independence was performed to investigate the relationship between BKS and student performance at the significant level of 5%.

One of the statistical techniques that is most frequently applied is regression analysis. Modeling the relationship between the explanatory and outcome variables is a crucial step in statistical modeling [10]. The most prevalent illustration of this is linear regression modeling, where the outcome covariate is a number. Instead, logistic regression analysis can be used if the outcome covariate is a binary response. Logistic regression is a common technique for modeling outcomes that fall into the range of 1 and 0. For this purpose, a logistic regression was performed to find adjusted odds ratio and crude odds ratio.

## Results

Sociodemographic information of the students is presented in Table 1. It can be seen that more than half (55.6%) of participants are female and the rest (44.4%) are males. The vast majority of the student age is between 18–24 as the typical university age followed by 6.25% aged between 25–30 and only 1.25 of the participants were more than 30. Furthermore, the vast majority (92%) of the students were single. University students (four years of study) participated by 91.6% and institute students (two years of study) were 18.4%. Similarly, the majorities (89.3%) of the students were from public universities and 14.7 were from private. Almost half of the students (48%) were from urban areas and 32.3 were staying at student accommodation and rural areas were 19.7%. The results also showed that the students come from low and medium and high income families were 4%, 67.8% and 28.2, respectively. Students’ smoking cigarettes were 7.2%, taking shisha 8.2% taking both cigarette and shisha were 2.1 and the remaining percentage (82.6%) they did not smoke.

**Table 1:** Sociodemographic information of the participants

Variables	Categories	No.	%
Gender	Male	986	44.3
	Female	1238	55.7
Age	18–24	2059	92.6
	25–30	139	6.2
	More than 30	25	1.2
Marital status	Single	2048	92.1
	Married	176	7.9
Study degree	University	1817	81.7
	Institutes	407	18.3
Study sector	Public	1897	85.3
	Private	327	14.7
Study level	First	590	26.5
	Second	730	32.8
	Third	392	17.6
	Fourth	473	21.3
	Fifth	24	1.1
	Sixth	15	0.7
Residence	Urban	1068	48.0
	Rural	440	19.8
	Student accommodation	716	32.2
BMI	Less than 18	162	7.2
	18–24.9	1530	67.8
	25–29.9	433	19.5
	More than 30	100	4.5
Parental education	Uneducated	478	21.9
	Primary	611	27.5
	Secondary	528	23.8
	University graduate	595	26.8
Income	Low	89	4.0
	Medium	1508	67.8
	High	627	28.2
Smoking	Cigarette	159	7.1
	Shisha	183	8.2
	Cigarette and shisha	46	2.1
	No smoking	1836	82.6

Table 1 illustrates a demographic overview of the participants. The majority of participants were female (1238, 55.7%). Out of 2,224 students, 2059 are aged between 18 to 24 years. Most of the participants were from first level (26.5%), second level (32.8%), third level (17.6%) or the fourth level (21.3%). Over 92% of participants were single and about 68% came from families of medium income families.

We have performed logistic regression models to find adjusted odds ratio and crude odds ratio. We have found that variables such as residence, BMI and income are statistically significant factors. The odds of BKS is reduced among students who live in accommodation by 54% (odds ratio = 54%, CI (41–71%), *p* value = 0.000). It seems that students with low income and normal or higher BMI are more likely to skip breakfast more regularly. The odds of BKS among students with BMI of 18–24.9 is reduced by 41% (odds ratio = 59%, CI (27–93%), *p* value = 0.027) and the odds of BKS is reduced among students with BMI of 25–29.9 by 45% (odds ratio = 55%, CI (31–95%). Additionally, students with medium or high incomes are more likely to skip breakfast as much as twofold in comparison with students with low income (medium income (odds ratio = 1.85, CI (1.08–3.17), *p*-value = 0.024), high income (odds ratio = 1.98, CI (1.12–3.51), *p*-value = 0.019). Lastly, student who are not smoking are 1.6-fold more likely to skip breakfast in comparison with students smoking cigarette.

The data in Table 3 show the relationship between BKS, sociodemographic information and academic performance. The statistical analysis showed that there is a significant relationship between academic performance and income (*P* = 0.000) and residency (*P* = 0.000). The data analysis showed that other sociodemographic parameters have no impact on academic performance and BKS. Therefore, it can be understood from Tables 2 and 3 that BKS is related to income, residency and then affects academic performance of the undergraduate students.

**Table 2:** Logistic regression analysis of breakfast consumption among university students

Variables	Categories	Crude OR	95%CL	*P*	Adjusted OR	95%CL	*P*
Gender	Male	1					
	Female	1.02	0.807–1.289	0.87	0.78	0.59–1.04	0.088
Age	18–24	1	Reference				
	25–30	1.067	0.65–1.74	0.796	0.84	0.49–1.43	0.511
	More than 30	Omitted					
Marital status	Single	1					
	Married	0.63	0.38–1.04	0.068	0.75	0.49–1.43	0.29
Study degree	University	1					
	Institute	1.07	0.79–1.46	0.651	1.04	0.43–1.29	0.81
Study system	Private	1					
	Public	0.64	0.44–0.93	0.02	0.71	0.48–1.06	0.09
Study level	First	1					
	Second	1.06	0.78–1.44	0.698	1.11	0.81–1.50	0.526
	Third	1.07	0.74–1.53	0.705	1.08	0.74–1.57	0.681
	Fourth	0.97	0.69–1.35	0.847	1.03	0.72–1.47	0.869
	Fifth	4.19	0.56–31.45	0.163	4.45	0.58–34.07	0.15
	Sixth	1.18	0.26–5.34	0.825	1.03	0.21–5.08	0.974
Residence	Urban	1					
	Rural	0.86	0.63–1.20	0.378	0.84	0.60–1.18	0.317
	Student accommodation	0.54	0.41–0.69	**0.000**	0.54	0.41–0.71	**0.000**
BMI	Less than 18	1					
	18–24.9	0.59	0.32–1.07	0.08	0.50	0.27–0.93	**0.027**
	25–29.9	0.55	0.31–0.95	**0.03**	0.51	0.29–0.88	**0.017**
	More than 30	0.63	0.29–1.37	0.245	0.52	0.23–1.14	0.102
Parental education	Graduate	1					
	Uneducated	1.12	0.80–1.57	5.11	1.36	0.95–1.94	0.096
	Primary	0.9	0.66–1.22	0.66	1.05	0.76–1.45	0.744
	Secondary	0.89	0.85–1.66	0.311	1.37	0.97–1.94	0.073
Income	Low	1					
	Medium	1.63	0.97–2.74	0.06	1.85	1.08–3.17	**0.024**
	High	1.82	1.05–3.15	**0**.**03**	1.98	1.12–3.51	**0.019**
Smoking	Cigarette	1					
	Shisha	1.02	0.60–1.75	0.93	0.92	0.53–1.61	0.83
	Cigarette and shisha	1.15	0.49–2.71	0.75	1.10	0.46–2.65	0.78
	No smoking	1.47	0.97–2.23	0.07	1.64	2.88–24.05	**0.000**

Bold values indicate significant effect/Profound effect

**Table 3:** The relationship between BKS and student academic performance

Variables	Categories	Low	Medium	High	*X*^2^	*P*
Gender	Male	101	529	354	4.792	0.091
	Female	103	710	427		
Age	18–24	186	1138	735	4.6657	0.323
	25–30	16	84	39		
	More than 30	2	17	7		
Marital status	Single	184	1147	717	1.4903	0.475
	Married	20	64	92		
Study degree	University	166	1012	639	0.0223	0.989
	Institute	38	227	142		
Study system	Public	179	1064	654	2.8177	0.244
	Private	25	175	127		
Study level	First	1	12	11	16.522	0.086
	Second	72	415	243		
	Third	45	229	118		
	Fourth	37	249	187		
	Fifth	204	1239	781		
	Sixth	0	7	8		
Residence	Urban	86	571	411	12.731	**0.013**
	Rural	41	250	149		
	Student accommodation	77	418	221		
BMI	Less than 18	11	89	61	9.8102	0.133
	18–24.9	145	832	553		
	25–29.9	34	262	137		
	More than 30	14	56	30		
Parental education	Uneducated	47	258	182	9.3986	0.152
	Primary	64	360	187		
	Secondary	44	286	198		
	University graduate	49	334	212		
Income	Low	22	44	23	72.636	**0.000**
	Medium	135	905	468		
	High	47	290	290		

Bold values indicate significant effect/Profound effect

Table 4 indicates relationship between breakfast consumption and using Chi-square test. According to the test there is no statistically significant relationship between skipping breakfast and BMI status (Chi-square = 9.11, *p*-value = 0.69). There is a statistically significant relationship between skipping breakfast and student’s income level (*p*-value = 0.000). BKS among students with BMI of 18–24.9 is reduced by 41% (odds ratio = 59%, CI (27–93%), *p* value = 0.027) and the odds of BKS is reduced among students with BMI of 25–29.9 by 45% (odds ratio = 55%, CI (31%-95%)We found that there are no relationship between students’ skipping breakfast and other factors such as age, gender, BMI, parental education, parental education, study level, study system and material status (Table 5).

**Table 4:** Shows the relationship between BKS and BMI

Skipping pattern	No.	%	BMI categories			
			UW*	N	OW	O
Never skip	626	28.15	50	120	426	30
1–2 times	623	28.01	49	129	415	30
3–4 times	405	18.21	33	80	275	17
5–6 times	237	10.66	14	40	174	9
Always skip	333	14.97	15	64	240	14

*UW* underweight, *N* normal, *OW* overweight, *O* obese

**Table 5:** Relationship between sociodemographic information among student and reasons for skipping breakfast

Variables	Categories	Time constraint	Lack of appetite	Breakfast not available	Not hungry	Afraid to be overweight	Other
Gender	Male	319	266	201	97	72	31
	Female	508	386	157	87	63	37
Age	18–24	775	600	333	168	124	59
	25–30	38	48	20	15	9	9
	More than 30	14	4	5	1	2	0
Marital status	Single	762	606	333	165	122	60
	Married	65	46	25	19	13	8
Study degree	University	686	517	296	154	110	54
	Institute	141	135	62	30	25	14
Study system	Public	707	549	320	145	121	55
	Private	120	103	38	39	14	13
Study level	First	216	180	78	50	45	21
	Second	282	205	122	61	39	21
	Third	140	119	67	36	21	9
	Fourth	178	136	83	33	27	16
	Fifth	7	7	6	1	2	1
	Sixth	4	5	2	3	1	0
Residence	Urban	400	373	92	108	51	44
	Rural	154	149	64	37	20	16
	Student accommodation	273	130	202	39	64	8
BMI	Less than 18	61	53	28	6	7	6
	18–24.9	576	447	253	132	79	43
	25–29.9	156	123	64	35	40	15
	More than 30	34	29	13	11	9	4
Parental education	Uneducated	160	150	103	36	27	11
	Primary	236	168	103	52	37	15
	Secondary	207	149	80	48	25	19
	University graduate	222	185	72	47	46	23
Income	Low	28	22	21	6	7	5
	Medium	567	439	251	116	89	46
	High	232	191	86	62	39	17
Smoking	Cigarette	52	42	40	18	5	2
	Shisha	60	56	37	14	11	5
	Cigarette and Shisha	16	10	11	2	7	0
	No smoking	699	544	270	150	112	61

**Fig. 1:**
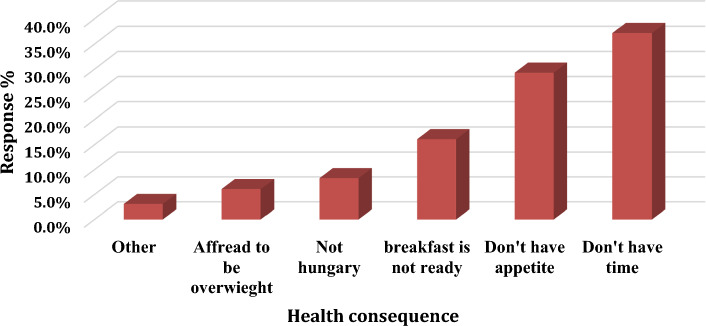
Frequency of reason for skipping breakfast

Figure 1 shows the reasons for skipping breakfast. The reasons include time constraint (37.2%), lack of appetite (29.3%), breakfast is not ready (16.1%), not hungry (8.3%) and afraid to be overweight (6.1%) Most of the male and female participants indicated that time constraints are the main reason for skipping breakfasts. The statistical data analysis showed that the main reasons provided by students with medium income are either time constraints or lack of appetite.

Table 6 indicates the frequency distribution of percentage of BKS and health consequence. Most of the students were felling hungry throughout the day while skipping breakfast (35.5%), felling tired throughout the day (19%) and lack of attention in the class (12%). Students thought that having snacks are unhealthy in comparison to skipping breakfast (51.8%) and over 24% of them had no snacks when they are skipping breakfast.

**Table 6:** Student’s frequency distribution in relation to BKS and health consequence and types of snacks

Variables	Categories	No.	%
Health status	Feeling hungry throughout the day	790	35.5
	Headache	218	9.8
	Stomachache	107	4.8
	Feel tired	422	19.0
	Luck of attention in class	266	11.9
	None of them	421	18.9
Type of snack	Healthy	468	22.4
	Unhealthy	1085	51.8
	No snacks	528	24.2

## Discussion

This study is among the early research studies investigating the impact of emergency period on BKS comparing to pre-emergency time. Pandemics like Covid-19 and its confinements and movement limitation can unfavorably affect eating behavior and lifestyle the community [5], particularly university students [6]. Regular breakfast is one of the most important factors to improve body well-being against several metabolic disorders then improving immune system and reduce risks of the pandemics [13].

The outcome of these study pointed out that the skipping of breakfast is prevalent. It is also obvious that income, BMI and residency have impact on the pattern of the skipping. A very few study have been conducted on this issue. Similar results were seen in earlier study when Japanese female students studied. The BKS were increased during the Covid-19 pandemic [27]. On the other hand, another Swedish study discovered that slight increased (0.2%) breakfast consumption was noticed among Swedish students [11]. The logistic regression analysis showed that the BKS is prevalent among normal and higher BMI students. This could verify the previous results stating connecting BKS with increasing weight [14].

Income also seems to be affecting BKS; higher income indicates higher BKS. This might due to the eating behavior where students are more comfortable eating snacks with counterparts students than with family. These results are similar to the previous study which found that students from higher family income skip breakfast [3]. They have attributed to the tendency of buying more ready-to-eat snacks particularly unhealthy snacks than breakfast due to time constraint as confirmed in Table 6. Similar to that students staying in accommodations skip more breakfast due to time constraint than students living with their families. This was also confirmed by previous Malaysian study [9].

The academic performance seems to be affected by BKS in relation to residency and income. In the results of this it was found that students who regularly skip breakfast can feel tired and less focused. This is particularly obvious in low income and students live on accommodation. This is especially seen in the case of Covid-19. This can be attributed to the fact that some of the classes were online and required more attention. When the students cannot pay enough attention they get lower marks. Another factor can be taking unhealthy snacks during Covid-19 as seen in the results that 51% of the students had unhealthy snacks and as it was reported previously that unhealthy snacks were more available during the pandemic [5, 15]. Previous study also stated that unhealthy snack intake negatively affects students’ academic performance [4].

The data have also showed that BKS causes negative health consequences in a short term at the top of them was feeling hungry throughout the day, followed by feeling tired and lack of attention in the class. It has been studied that missing meals or fasting can cause hypoglycemia and trigger headache [26]. An earlier study found that BKS is associated with tiredness and poor attention in the class and lower academic performance [1]. It is generally accepted that BKS impairs cognitive function and consequently academic performance [8]. On the other hand, it was stated that consuming breakfast is associated with a better health [12].

The data analysis indicated that the time constraint is the most common reasons for skipping breakfast along with other reasons including lack of appetite and breakfast unviability. These results are in congruence with those by [2, 3, 17]. These reasons might be due to the fact the many students stay and eat late night [16]. Consequently, they do not awake early and prepare breakfast as well as not have appetite to eat.

It is worth mentioning that the main limitation of the study is conducting it online due to the movement restriction and being able to interview students face to face. There was also another factor including psychological parameters that could be studied and might interfere with other parameters.

## Conclusion

The aim of present study was to study breakfast skipping frequency, factors associated with, health consequence and undergraduate students academic performance during Covid-19 pandemic as earliest studies focusing on this area. It has been found that factors associated with skipping breakfast are BMI, type of accommodation, and income level. Not having time and not having an appetite were the most common reasons for skipping breakfast. The consequences of skipping breakfast were feeling hungry throughout the day, feeling tired, and not paying attention in class and low academic performance. We also found that having unhealthy snack to compensate skipped breakfast is common. It is important that students follow a healthy diet and life style in order to avoid the negative consequences of pandemics and gain better academic performance.

## Data Availability

No datasets were generated or analyzed during the current study.
